# A Comprehensive Treatment Approach in a Patient With a Malignant Peripheral Nerve Sheath Tumor: A Case Report

**DOI:** 10.7759/cureus.31136

**Published:** 2022-11-05

**Authors:** Geetika Malhotra, Anurag Bhattacharjee, Tushar Nagtode, Pankaj Gharde

**Affiliations:** 1 Department of General Surgery, Jawaharlal Nehru Medical College, Datta Meghe Institute of Medical Sciences, Wardha, IND

**Keywords:** neck tumor, malignant tumor resection, neurofibromatosis 1, schwannoma, case report, sarcoma, malignant peripheral nerve sheath tumors

## Abstract

Malignant peripheral nerve sheath is a rare neoplasm seen in humans, with a very small percentage of occurrence in the cervical region. It is an aggressive form of sarcoma, usually arising from peripheral nerves. In this case study, we present a 61-year-old male who was reported to the hospital with a chief complaint of swelling on the right side of his neck. Magnetic resonance imaging (MRI) revealed a heterogeneous mass in the cervical region, which is reported as a schwannoma. The patient was posted for surgery for tumor excision. The patient was managed with analgesics, physical therapy, and wound care. The patient's symptoms were relieved, and he was discharged. In conclusion, combined surgical resection and adjuvant treatments like physical and medical therapy improved the functional outcome. However, this literature does not address radiation therapy.

## Introduction

Malignant peripheral nerve sheath tumor (MPNST) is a connective tissue tumor, and because of its appearance and origin, it falls under the category of sarcomas. The most common presentation includes numbness, dizziness, and edema, with or without pain in the affected limb. It may also reduce joint mobility. Although the pathophysiology of MPNST is unclear, it has a strong genetic association due to the high prevalence in both the first and second-degree relatives of the patient. [1,2]. About half of the patients have neurofibromatosis, which is an established underlying cause of MPSNT. Patients with neurofibromatosis type 1 have an 8%-13% higher risk of developing MPSNT than the general population. Loss of sequence from the 17q arm of the chromosome with complete inhibition of the neurofibromatosis-1 (NF-1) gene is the pathogenesis of MPNST associated with neurofibromatosis [3]. The diagnosis of this condition is done radiologically using magnetic resonance imaging (MRI). MPSNT is also known as neurofibrosarcoma. Treatments for neurofibrosarcoma are similar to those for other sarcomas, including symptomatic treatment, chemotherapy, surgical excision, and/or radiotherapy. The overall five-year survival rate for people with MPNST is between 23% and 69 %. Excision of the tumor and surrounding tissue is critical for a better prognosis. Radiation therapy can be used before and after surgery. Surgery is often followed up with radiation therapy of the resected area to reduce the likelihood of the recurrence of an isolated or localized tumor [4].

## Case presentation

**Figure 1:**
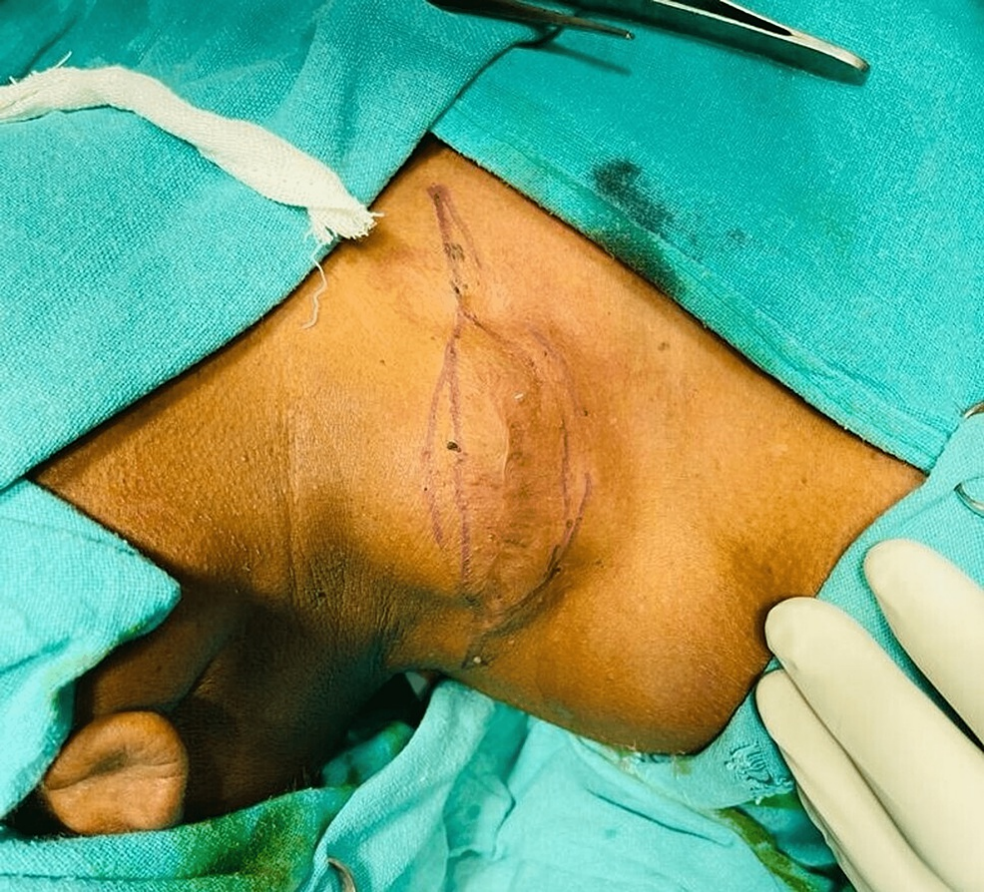
A firm swelling measuring 6x5 cm on the right side of the neck posterior to the sternocleidomastoid muscle.

A 61-year-old male reported to the outpatient department with a complaint of swelling in the right side of the neck for the past six months, which was painful during neck movements. On examination, there was a 6x5 cm firm swelling on the right side of the neck with no local rise in temperature and no overlying skin changes. The swelling was non-tender and non-mobile, with no other palpable cervical lymph nodes. The swelling was fixed to the underlying tissue and the skin. It was without any discharge or bleeding (Figure 1).

**Figure 2:**
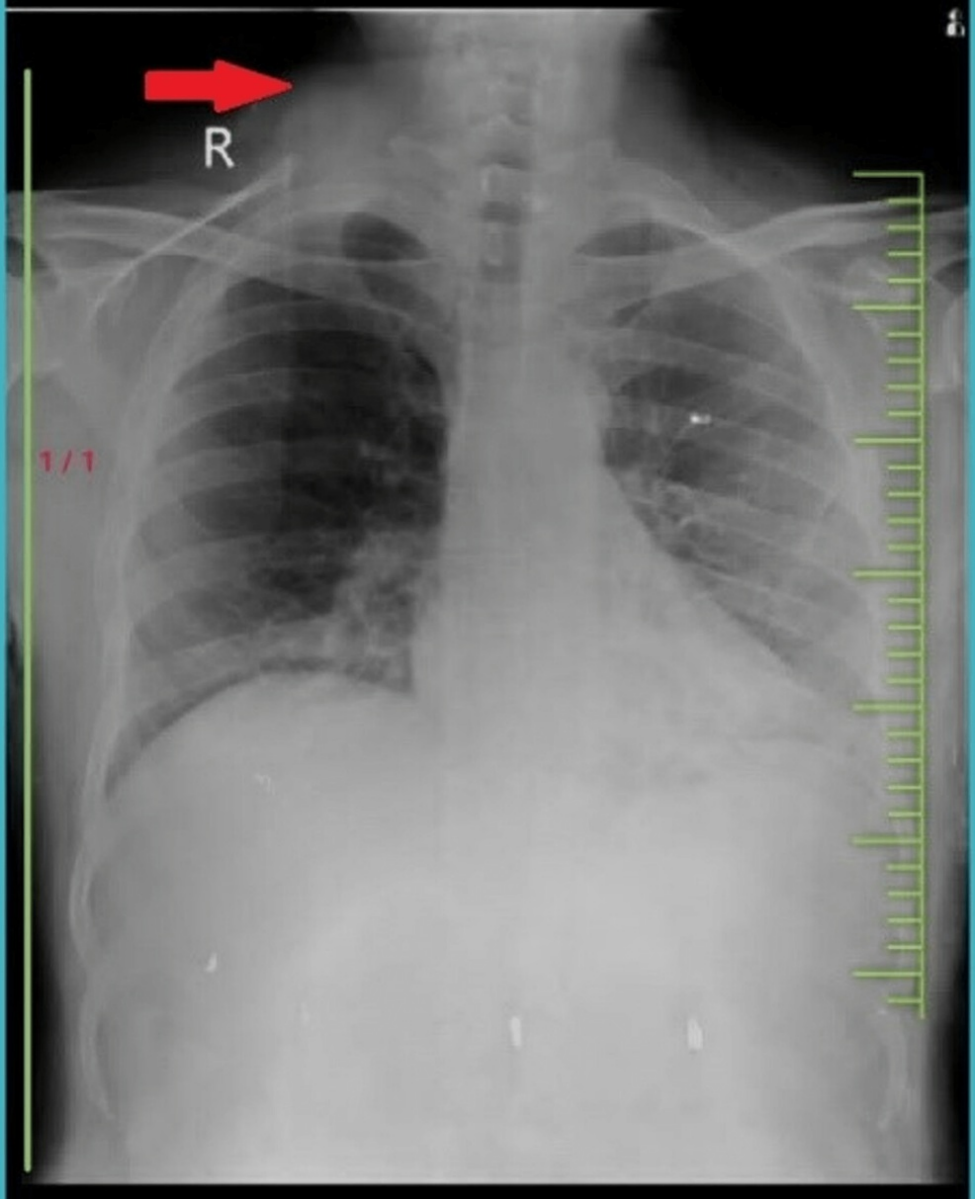
X-ray showing a mass in the right supraclavicular region with no bony involvement.

Plain radiographs of the chest and neck showed a mass lesion in the deep soft tissue plane of the right supraclavicular region, most likely suggestive of a mass in the right supraclavicular region with no bony involvement (Figure 2).

**Figure 3:**
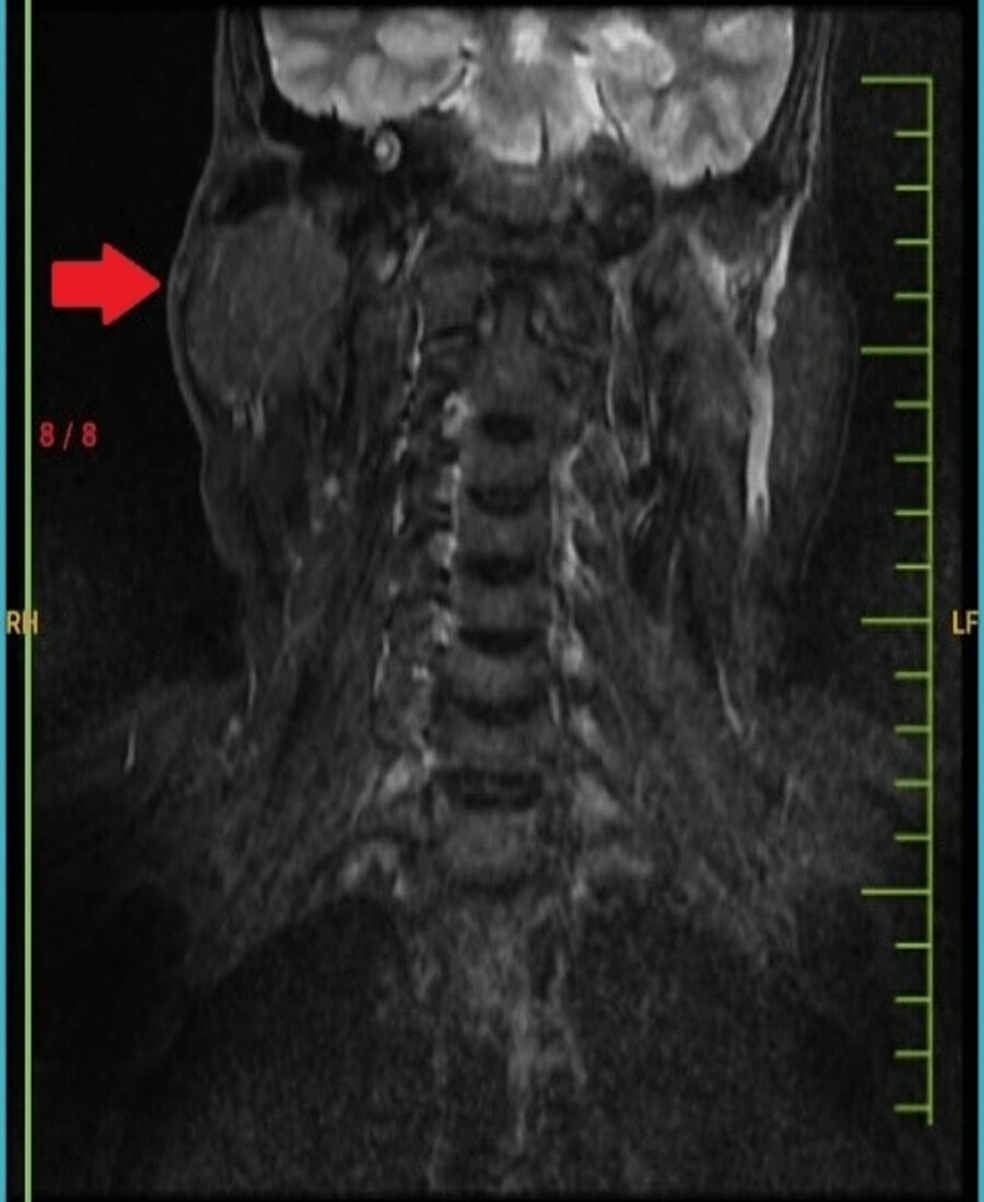
An MRI of the posterior cervical region with an arrow showing enhanced mass.

Magnetic resonance imaging (MRI) revealed a heterogeneously enhancing lesion with areas of necrosis in the deep soft tissue plane of the right supraclavicular region. It measured 6.2 x 5.2 x 4.5 cm in size and was heterogeneously hyperintense on T2WI film and hypointense on T1WI film. Superiorly, the mass lesion was seen posterior to the sternocleidomastoid muscle, causing its anteromedial displacement. Inferiorly, the lower pole of the lesion was seen posterior to the clavicle. The fat planes with surrounding structures were maintained. The tumor mass was vascular with no capsular invasion. This was suggestive of a soft tissue tumor, with differentials being schwannoma, neurofibroma, leiomyosarcoma, and a dermoid cyst (Figure 3).

**Figure 4:**
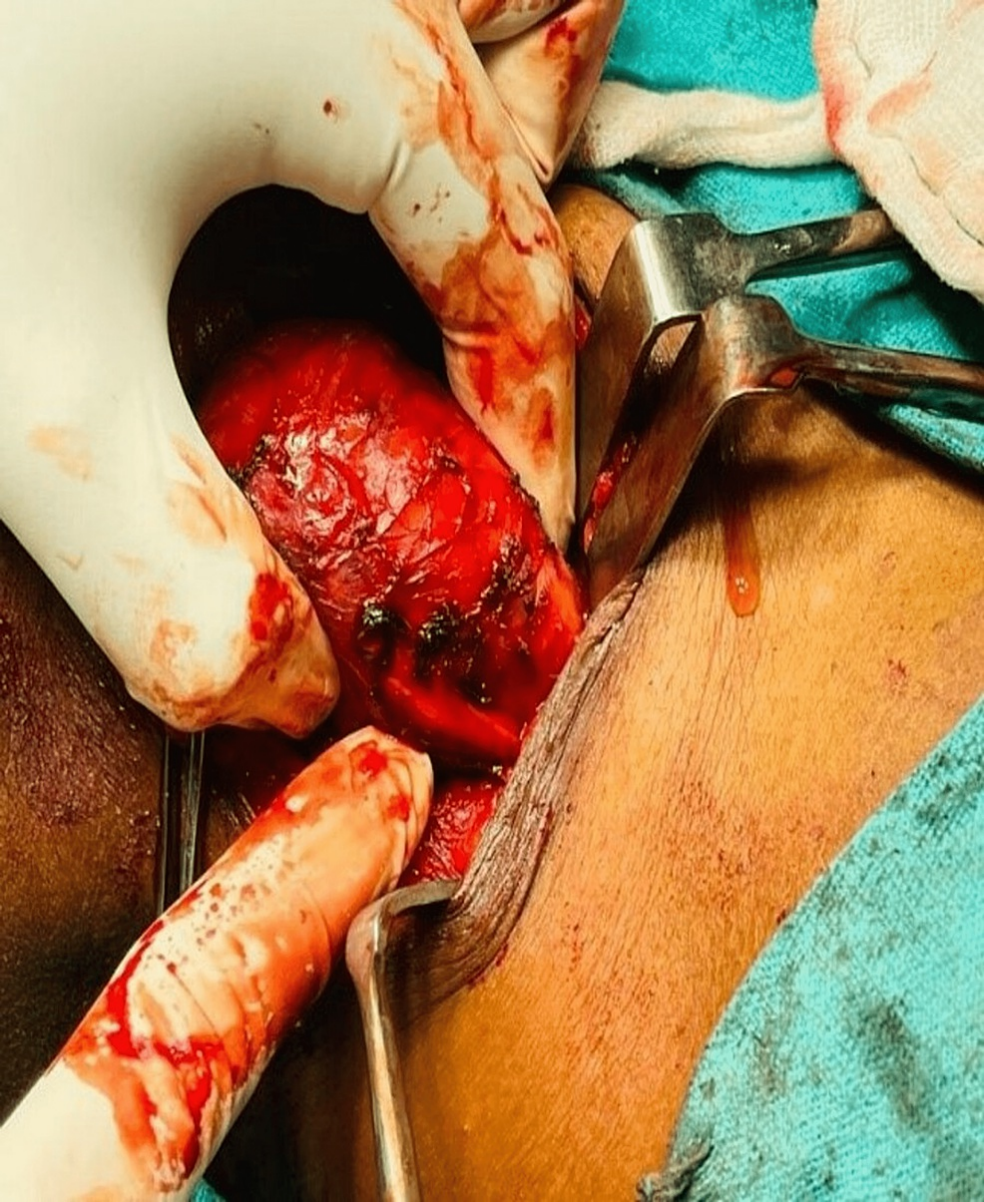
Neck dissection showing a mass of approximately 6x5 cm.

**Figure 5:**
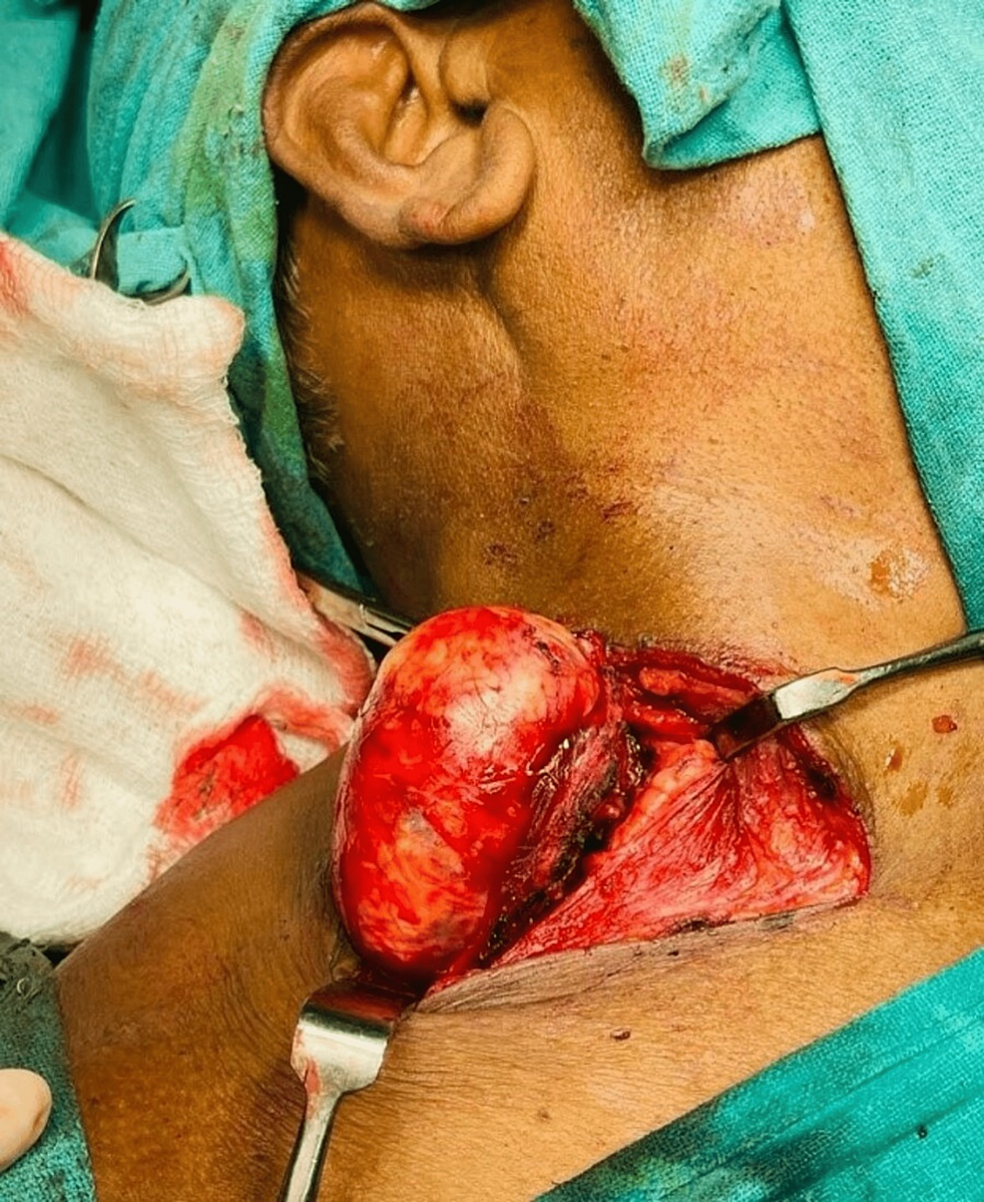
The removal of the malignant nerve sheath tumor.

**Figure 6:**
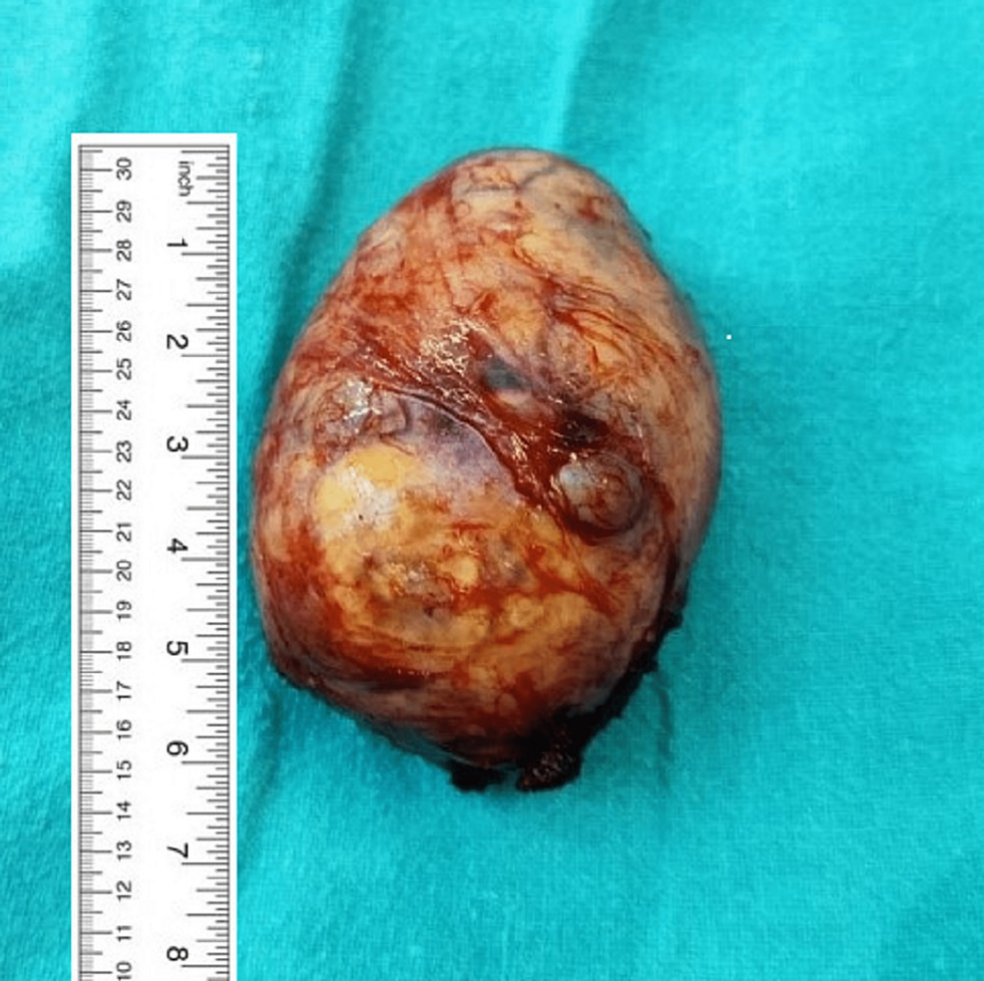
The specimen of the removed nerve sheath tumor.

Fine-needle aspiration cytology (FNAC) was done, and the patient was diagnosed as having a malignant peripheral nerve sheath tumor. Surgical excision of the mass was planned. The patient had no comorbid conditions, was an American Society of Anesthesiologists (ASA) class 1 patient, and was fit for surgery. The patient was positioned under all aseptic precautions, with parts painted and draped. An elliptical incision was made over the right-sided neck over the Langer line (previous scar site included), measuring 4 cm. Incisions were made, and the platysma was divided. The neck mass was identified and found to be adherent to surrounding structures. A blunt dissection was done surrounding the mass. The base of the mass was separated with bipolar cautery. Proper hemostasis was achieved. Drain No. 14 was placed, and the incision was closed in layers. A sterile dressing was applied, and the procedure was declared uneventful (Figures 4-6).

Postoperatively, the patient was extubated and shifted to postoperative recovery with no intraoperative complications. The patient was managed with analgesics, daily dressings, and daily physiotherapy. The patient was discharged after seven days of surgery, with the advice to follow up in the OPD next week for complete suture removal. On follow-up, the patient was hemodynamically stable with no fresh complaints. The histopathology report was suggestive of a schwannoma.

Table 1 shows the timeline of events.

**Table 1:** The timeline of events.

Event	Date
Admission to the hospital	19/09/2022
MRI and required investigations	20/09/2022
Surgery	28/09/2022
First follow-up	12/10/2022

## Discussion

Malignant peripheral nerve sheath tumor (MPNST) is a recurrent mutation of polycomb repressive complex 2 (PRC 2), embryonic ectodermal proteins (EED), and suppressor of zest 12 homolog (SUZ 12). This mutation causes altered chromatin, which promotes MPNST malignancy [5]. The occurrence of MPNST is 45% in the extremities, 34% in the trunk, and 19% in the neck area, which is the least common. The prognosis of the disease is poor due to the complications of surgery during the resection of the tumor. Depending on the size, location, and structures in the vicinity of the tumor, this condition has a high risk of sarcoma-specific death [6]. The disease has a high rate of recurrence after resection. Large tumors and positive surgical margins are the significant factors for local recurrence, while the factors responsible for distal recurrence were tumor size and grade [7-9]. The best available option for treatment includes a surgical resection followed by radiotherapy and chemotherapy [8,10].

Chemotherapy as an adjuvant has been opted for in cases with tumor sizes greater than 5 cm and high-grade MPNST, irrespective of the tumor site. However, recent studies have shown that patients who opt for chemotherapy without surgery have the worst prognosis and lower survival rates; the same results have been cited in many other studies [11,12]. In light of the strong association with neurofibromatosis I and the difficult differential diagnosis of other soft tissue tumors, emphasis should be put on excluding further manifestations of neurofibromatosis I and secondary tumors.

In this case study, we expect a positive outcome compared to the available literature as the surgical margins were negative and we opted for daily physical therapy for functional improvement in the shoulder and respiratory function of the patient. Even though we do not have a five-year post-treatment result, the surgical approach and the adjuvant therapy will have promising results.

## Conclusions

Malignant nerve sheath tumors are uncommon, particularly those affecting cranial nerves. In our case, there was no involvement of the capsule and surrounding structures; hence, it was easy to remove, and a pre-operative FNAC guided the treatment plan. A thorough neurological examination also aids in this. Suspicion of neurofibromatosis should be strong in cases with malignant peripheral nerve sheath tumors. As this remains a very rare neoplasm, the negative surgical margins and adjuvant physical therapy have improved the condition and relieved symptoms in our patient. A close postoperative follow-up is mandatory to eliminate recurrence. The available literature has not portrayed the impact of chemotherapy and radiotherapy on the condition. This treatment strategy has improved the functional status in our case. A protocol needs to be established for managing these tumors.

## References

[1] Strike SA, Puhaindran ME (2019). Nerve tumors of the upper extremity. Clin Plast Surg.

[2] James AW, Shurell E, Singh A, Dry SM, Eilber FC (2016). Malignant peripheral nerve sheath tumor. Surg Oncol Clin N Am.

[3] Mohamad T, Plante C, Brosseau JP (2021). Toward understanding the mechanisms of malignant peripheral nerve sheath tumor development. Int J Mol Sci.

[4] Jones J, Cain S, Pesic-Smith J, Choong PF, Morokoff AP, Drummond KJ, Dabscheck G (2021). Circulating tumor DNA for malignant peripheral nerve sheath tumors in neurofibromatosis type 1. J Neurooncol.

[5] Korfhage J, Lombard DB (2019). Malignant peripheral nerve sheath tumors: from epigenome to bedside. Mol Cancer Res.

[6] Stucky CC, Johnson KN, Gray RJ, Pockaj BA, Ocal IT, Rose PS, Wasif N (2012). Malignant peripheral nerve sheath tumors (MPNST): the Mayo Clinic experience. Ann Surg Oncol.

[7] Anghileri M, Miceli R, Fiore M (2006). Malignant peripheral nerve sheath tumors: prognostic factors and survival in a series of patients treated at a single institution. Cancer.

[8] Stucky CC, Johnson KN, Gray RJ, Pockaj BA, Ocal IT, Rose PS, Wasif N (2012). Malignant peripheral nerve sheath tumors (MPNST): the Mayo Clinic experience. Ann Surg Oncol.

[9] Abdel Al S, Abou Chaar MK, Asha W, Al-Najjar H, Al-Hussaini M (2020). Fungating malignant peripheral nerve sheath tumor arising from a slow-growing mass in the forearm: a case report and review of the literature. J Med Case Rep.

[10] Ferner RE, Gutmann DH (2002). International consensus statement on malignant peripheral nerve sheath tumors in neurofibromatosis. Cancer Res.

[11] Voorhies J, Hattab EM, Cohen-Gadol AA (2013). Malignant peripheral nerve sheath tumor of the abducens nerve and a review of the literature. World Neurosurg.

[12] Zou C, Smith KD, Liu J (2009). Clinical, pathological, and molecular variables predictive of malignant peripheral nerve sheath tumor outcome. Ann Surg.

